# Nudging digital physical activity breaks for home studying of university students—A randomized controlled trial during the COVID-19 pandemic with daily activity measures

**DOI:** 10.3389/fspor.2022.1024996

**Published:** 2022-11-24

**Authors:** Monika Teuber, Daniel Leyhr, Juliane Moll, Gorden Sudeck

**Affiliations:** ^1^Faculty of Economics and Social Sciences, Institute of Sports Science, University of Tübingen, Tübingen, Germany; ^2^Methods Center, Faculty of Economics and Social Sciences, University of Tübingen, Tübingen, Germany; ^3^Interfaculty Research Institute for Sports and Physical Activity, University of Tübingen, Tübingen, Germany

**Keywords:** physical activity breaks, digital nudging, home studying, university students, intervention, motivational prompts

## Abstract

University students are of particular public health interest because they are at high risk for physical inactivity and sedentary behaviors. In conjunction with the COVID-19 pandemic, sedentariness and physical inactivity were reinforced, as the pandemic led to an increase in home studying. Physical activity (PA) breaks have been identified as promoting factors for university students' physical and mental health. Therefore, the present study explored an approach to nudge students to take PA breaks at home while studying. The purpose was to test the effectiveness of digital nudging for PA breaks for 10 days using a randomized intervention design during the COVID-19 pandemic. It included an intervention group who received daily digital motivational prompts for PA break videos and a minimal intervention control group who got low-level access to PA break videos *via* a one-time link sent to the media library. Using a sample of university students in the southwest of Germany (*n* = 57), two-level binary logistic regression models were calculated to predict daily participation in PA breaks during the intervention period depending on the nudging intervention, as well as previous participation in PA breaks, the general PA level of the subjects before the intervention, the time spent on PA and on home studying in a day, the kind of day during the intervention (weekday vs. weekend), and the students' age. Results revealed that the digital nudging intervention did not show any significant effect on the likelihood to participate in PA breaks on a given day (0.69 ≤ β ≤ 0.75, *p* > 0.3). Instead, an individual-level effect revealed that the longer a student studied at home over the course of a day, the more likely he or she was to take a PA break (1.07 ≤ β ≤ 1.11, *p* < 0.001). Current findings show that individual characteristics such as daily time spent on home studying, which can change over the course of the intervention phase, are relevant considerations within nudging intervention in university setting. This provides initial insights especially for digital PA breaks for students during home studying.

## Introduction

University students are of particular public health interest because they are at high risk for physical inactivity and sedentary behaviors. The transition from school to university often marks a particular risk for becoming physically inactive (1). Increasing academic workload with its resulting problems in time management regarding work and social demands results in students' physical inactivity (2). Additionally, in Germany, students show the highest percentage of sedentary time among all working occupational groups (3). This is determined by critical factors of the university setting such as sitting in classes, self-study learning, and smartphone usage (4, 5). Technological advances allowing students to study in the comfort of their own homes without changing locations, as well as higher screen time have also led to an increase in sedentary behaviors (6, 7).

As a result, the benefits of health-enhancing physical activity (PA) are less likely to be achieved and the health risks of sedentary behaviors increase (8). This is of particular relevance for university students, as they often face widespread health problems associated with the high demands of academic studies. For example, university students suffer more often from perceived stress (9) and report physical and psychological complaints more often than their peers (9–11). Furthermore, long sitting during lectures results in increased fatigue and lower concentration (12, 13). Given the demands of academic studies, PA can contribute to ensuring the cognitive functioning of young adults. Research has shown that PA can improve learning outcomes by activating brain areas relevant to learning (14), promoting synapse formation, supporting neuronal cell regeneration and growth (15), and stimulating cardiovascular circulation and thus increasing the supply of oxygen to the brain (16). Taken together, current PA recommendations on international and national levels (17, 18) particularly apply for university students as they give advice to reduce sedentary behavior [e.g., avoid long, uninterrupted periods of sitting and, if possible, to regularly interrupt sitting with PA (17)] and to achieve sufficient levels of moderate-to-vigorous PA to gain the various health benefits.

Particularly during the COVID-19 pandemic, sedentariness and physical inactivity were reinforced. PA among young adults was observed to decrease compared to pre-pandemic levels, whereas sedentary behavior further increased (19–23). An almost world-wide quarantine was ordered in Spring 2020 in order to reduce the incidence of infection. This led to an increase in the utilization of home office for work, schools and universities and a reduction in leisure possibilities such as doing sports or meeting friends. Consequently, the COVID-19 pandemic functioned as a kind of driver for physical inactivity and sedentary behavior. For example, nearly half of an Australian adult sample reported a reduction in PA (21), and among a sample of young adults in Hong Kong, 70% reported that they had reduced their PA (22). Regarding sedentariness, Spanish university students, for example, increased their sedentary time by more than 50%, including leisure-time and study-related screen time (23). Therefore, it is even more important to promote PA in everyday life in order to minimize sedentary behavior along with its risks, especially during this unique period of the COVID-19 pandemic, where home studying and social distancing characterized study life.

Breaks that involve PA and interrupt sedentary behavior have been identified as promoting factors for university students' physical and mental health. Different approaches are possible for the implementation of PA breaks. For example, PA breaks that are guided by an instructor in an in-person session in presence lead to better cognitive functioning and learning behavior (24, 25) like better classroom behavior in learning settings (e.g., time on task or involvement) (26–28), better task-related participation behavior (29) or short-term effects on cognition tasks (30). Regarding the acute effects of PA breaks, higher attention and cognitive performance, improvements in the level of interest, as well as improved mood ratings have been observed (13). Moreover, compared to no break and psycho-regulatory breaks, PA breaks show greater effects on students' vigor and regenerative ability regarding tension and fatigue caused by cognitive load. There is also evidence that participants who attended PA breaks in classroom settings acquired a higher amount of PA level measured *via* daily step count average with pedometers compared to participants who did not attend (6).

Nudging could be another useful method and approach in promoting PA breaks, particularly if the breaks are not able to be instructed in person. Nudging is a method of influencing people's behavior without resorting to prohibitions and commands (31). It can take place both consciously and subconsciously. Different forms of nudging that can promote PA have been discussed recently (32). Information disclosure is a common way of consciously nudging. Providing general information, health benefits for example, can promote awareness of actions (33, 34). Warnings in the form of disclosing risks also encourages people to reflect on and change existing behaviors (34). Furthermore, the identification of an issue leads to a higher likelihood of behavioral implementation. For this reason, appealing to intentions is also an effective way of nudging (34).

During periods of extensive home studying, digital ways of transmitting nudging are especially needed to reach students. Inactivity in individuals is often due to forgetfulness, procrastination or lack of time. Reminders *via* push messages or emails can prompt action. Prompting frequent short PA breaks may be one effective way to increase PA and reduce sedentary behavior (35). Research on digital media as transmitters of nudges has revealed first insights on positive effects of low-threshold interventions, such as daily emails or on-screen break prompting systems (36–38) as well as digital personalized applications with exercises, and advanced information (39, 40). These insights showed for example that people adhere to the email or SMS message encouragements for daily stair-walks at work (36, 41, 42), or for health enhancing website use (37). They also adhere to digital application for break up sedentary behavior at workplaces (40). Other findings revealed that nudging can be improved *via* personalization like individual preferences or the option to sync prompts with online calendars (39). However, the current state of research focuses on digital nudging interventions at workplaces and still leaves open questions on how sitting times in the university setting could be reduced by PA breaks specifically for students during home studying. Additionally, since student-life has changed drastically with the COVID-19 pandemic and most of the field studies addressing PA breaks (6, 13, 24, 43–45) took place beforehand (13, 29, 46), little is known about PA breaks not instructed in person during home studying.

The aim of the present study is to compare different approaches on how to reach students in order to promote PA breaks during home study times. Therefore, two different ways of accessing and promoting the use of video-guided PA breaks are compared. The main focus is on a digital nudging intervention which includes daily digital motivational prompts for PA breaks with one integrated PA break video and further links to a video portal that provides numerous PA break videos using a team learning software tool that is highly accessible for the given university students. This digital nudging intervention will be compared to a minimal-intervention control group that did not get such daily nudges but rather only received low-level access to the same video portal with PA break videos.

The primary outcome addresses daily participation in PA breaks during the intervention period of 10 days, which is expected to be higher in the digital nudging condition when including daily motivational prompts. Picture messages should stimulate a reflexive process following the reception and absorption of information and encourage participation in PA breaks. Thereby, issues of increasing PA and reducing sedentary behavior, recovery, or taking breaks in general were addressed to provide health information, disclose risks, and reach the active identification of students. Accordingly, the research question aims to investigate whether the selected digital nudging intervention has a beneficial effect on taking PA breaks during home study periods. Additionally, in terms of secondary effects and controlling for confounders for the main hypothesis, the study considers other parallel mechanisms which might influence daily participation in PA breaks. Namely previous participation in PA breaks, the general PA level of the subjects before the intervention, the time spent on PA on a day during the intervention phase, the time spent on home studying on daily level, the kind of day during the intervention (weekday vs. weekend), and finally age as a sociodemographic factor is additionally considered for the study.

## Methods

### Study setting

During the implementation of the study, containment measures associated with the COVID-19 pandemic did exist. Home studying and digital learning characterized study life and a so called “digital semester” was in effect at the University of Tübingen due to the COVID-19 pandemic. Courses were mainly to be taught online—synchronous, i.e., live, and asynchronous, for example, *via* a recorded lecture. Regarding leisure time, there were contact restrictions (social distancing), the performance of sports activities in groups were not permitted, and sports facilities were closed. The university sports department was also not allowed to offer face-to-face sports activities, but only online activities.

This first-time digital summer semester 2020 was the starting point for the introduction of the new digital PA break offer “Bewegungssnack digital” [in English “exercise snack digital” (ESD)], which provided digital PA breaks for everyday home studying. This offer was accompanied by a scientific evaluation in the follow-up digital semester, which was the basis for the present study. The ESD intended to support students in home studying by promoting regular PA breaks. The ESD offer consisted of 5–7-min videos with guided physical exercises and health-promoting explanations for a PA break. They were categorized into three thematic foci: activation, relaxation, and coordination. Within the videos, the exercises were demonstrated by one or two student exercise instructors. Additionally, descriptions of the relevant execution features of each exercise were displayed in the video *via* textual cues.

### Design and procedures

This randomized intervention study included an intervention group (IG with digital nudging for the PA break video ESD) and a minimal-intervention control group (MICG with access to the PA break video offer *via* a one-time provision of the link to the video portal).

**Figure 1:**
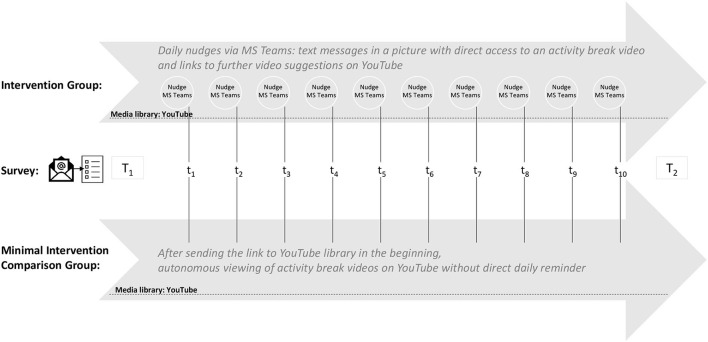
Design and procedure of the study for both groups: digital nudging intervention group (IG) and minimal intervention control group (MICG).

Data were collected before (T1) and after the intervention (T2) as well as daily during the intervention phase. During the ten-day intervention phase (Wednesday–Friday), daily surveys (t_1_-t_10_) were conducted in both groups, which were sent by email at 7 p.m. every evening. Subjects were asked to answer questions about their home studying behavior. They were also asked to answer a questionnaire one– two days before and after the intervention phase (Figure 1). The surveys were implemented online *via* the software UNIPARK. The recording and evaluation of the data were anonymized. Each participant used a personal code under the guidance of certain questions that only the participant himself/herself had created and knew.

The study researchers who created the intervention in the form of daily digital nudges to use the digital PA break offer were aware of the assignment of the conditions. They were the same people who mailed the questionnaires and statistically analyzed the collected data from the surveys. The exercise instructors and video designers of the digital PA breaks, on the other hand, were not informed about the conditions of the study in detail, but only knew the goal of the PA breaks and the use in their everyday university life and studies.

Participants were recruited *via* different digital ways: The study was advertised to students of the University of Tübingen *via* a circular email to those who registered for the digital PA breaks course offer on the homepage of the university sports department, and also to all students of the University of Tübingen *via* the university email distribution list. In addition, advertisement on social media of the university sports department of Tübingen (Facebook, Instagram, YouTube, homepage) took place. The study was announced using the incentives of one i-Pad, two smart watches and five tablets that were raffled off to participants who fully participated in the entire intervention.

Subjects were informed that participation in the study was voluntary and that no personal disadvantages would result from non-participation. The written informed consent was obtained together with the approval of the data protection regulations directly in the first questionnaire (T1) by means of a mandatory selection field. The faculty of the University of Tübingen of the first authors institution had given a positive ethical approval for the study.

### Participants

**Figure 2:**
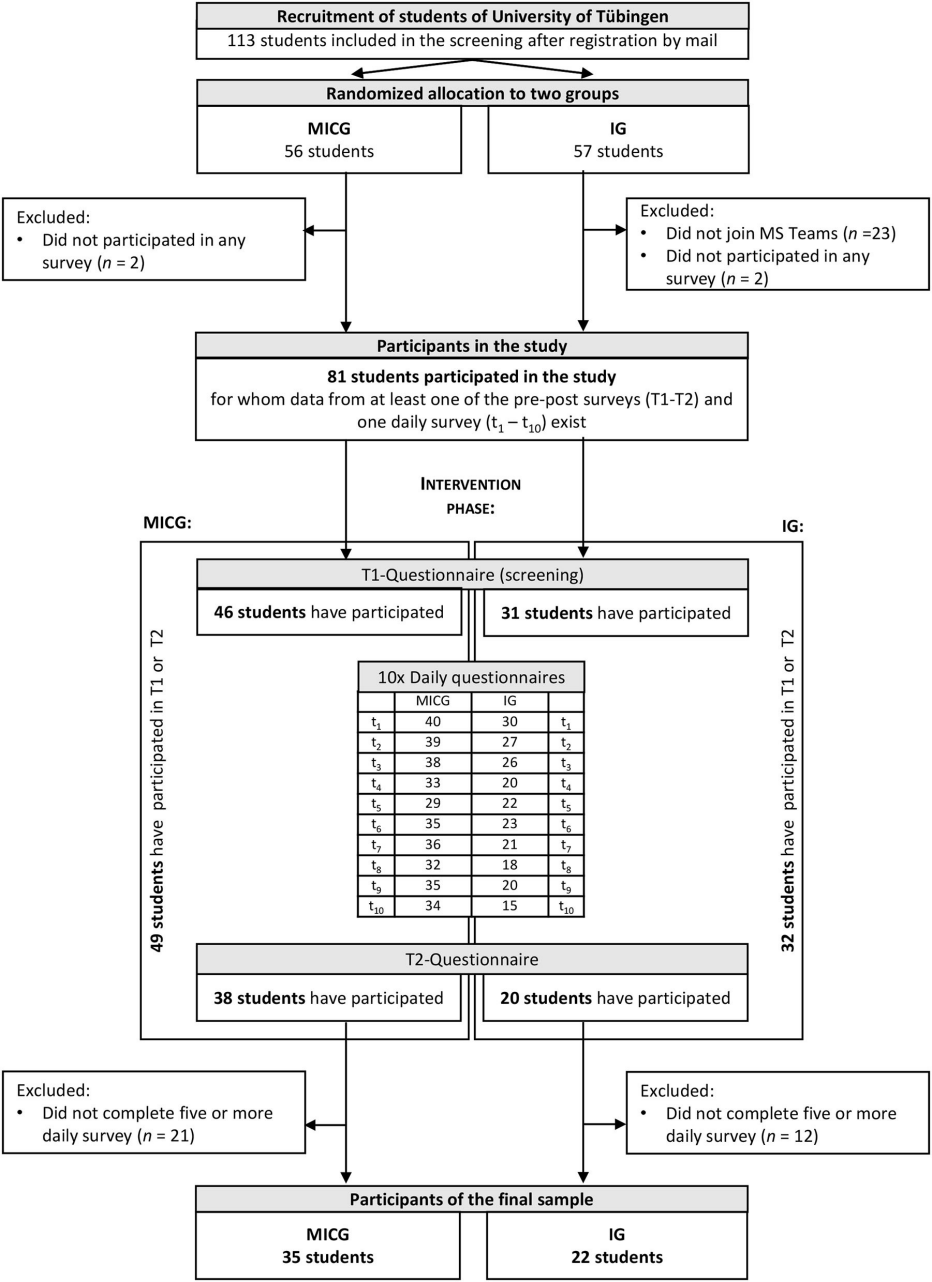
Flow diagram of the study participants (MICG, minimal intervention control group; IG, intervention group).

The study group consisted of students from the University of Tübingen in the Southwest of Germany. Initially, 81 participants (male = 11, female = 65, diverse = 1, not stated = 4) took part in the study. However, the number of participants in the final sample dropped to *N* = 57 (male = 6, female = 47, diverse = 1, not stated = 3) because only subjects who had participated in the daily surveys on at least half of the days of the intervention period were included (Figure 2). These participants were between 18 and 32 years old (*M* = 23.52, SD = 2.81) and were studying in their 1st to 13th semester (*M* = 5.76, SD = 4.11). Participants studied one of the following major courses of study: mathematical-scientific majors (34.0%), social science majors (22.6%), philosophical majors (18.9%), medicine (13.2%), theology (5.7%), economics (3.8%), or law (1.9%). For 5 days a week on average (SD = 1) they studied at home and had half a day (SD = 1.5) a week on average of on-site classroom teaching on university campus. Most of them lived in a residential community (46.3%), followed by living together with their partner (33.3%), together with their family (16.7%), and living alone (3.7%). In the beginning of the study, more than a half of the students (55.6%) met the national physical activity recommendations for health-promoting PA, as indicated by applying the PA questionnaire of the European Health Interview Survey (see below).

Thirty-five participants belonged to the MICG (male = 3, female = 30, diverse = 0, not stated = 2) and 22 participants to the IG (male = 3, female = 17, diverse = 1, not stated = 1). The IG had to complete an additional step (join the platform MS Teams) before the study began.

### Intervention

The intervention phase was realized in the middle of the lecture period between 25th of November and 4th of December 2020. In the whole study, subjects of both groups, IG and MICG, were asked to continue with their normal home study routine. They were also asked to perform ESD at any time in their daily home study routine, which could also be implemented online, at home or at any other location. Although both groups differ in the kind of direct reminders for ESD, it is important to note that both groups received indirect reminders due to the daily questionnaire in the evening that they were participating in a study that involved taking PA breaks. The difference in information and access to the digital PA break offer ESD between both groups are described in the following subchapters.

#### IG: Digital nudging intervention

Previously, and after randomized assignment to IG, participants had to create an account on the software MS Teams and join the team using the team code sent to them. MS Teams was chosen because it was one of the leading platforms for online teaching at the University of Tübingen, and every student had access to the program. Participants who joined the team folder of MS Teams got motivational nudges sent from it once a day with direct video access to ESD.

Nudges sent daily at 10:30 a.m. during the intervention phase including picture messages encouraging participation in the ESD following three categories:

1. Increasing PA and reducing sedentary behavior: For example, the nudges in this category included information on exercise recommendations, motivation to move or the risks of sitting.
2. Recovery: In this category, the focus of the nudges was on the recovery effect of breaks and the reduction of complaints. For example, the nudges addressed the need for rest and emphasized the positive effect on performance.
3. Break: The nudges included general information on how to take a break, such as the right time or duration.

In the present study, conscious variants of nudging were chosen, through which students knew which behavior should be influenced. Nudges were transparent and can be classified as type 1 according to the model of Heidbrink and Klonschinksi (47). They attract attention, whereby the action is only implemented after a reflexive process after encouraging the absorbtion and reflection of information as well as corresponding actions. According to the “EAST-Framework” (Easy, Attractive, Social, Timely) of the Behavioral Insights Team of the British Government (48), certain principles were taken into account in the designing of the nudges (Supplementary Figures 1, 2):

“*Make it simple*” (48): For a nudge to have the desired effect, it is important that it is as simple as possible. This means that the information is easily accessible and easy to understand. In this study, this factor was realized *via* push picture messages which show up on the screen as well as direct access to the video-data and further links directing to three more videos. The push messages were graphically designed with short, simple sentences.

“*Make it attractive”* (48): For a nudge to be considered, it is important that it attracts attention. Attractive content design, oriented toward the target group, is a key point here. The nudges in this study realized this point of attractiveness by relying on students' long sedentary behavior and stress involved in studying.

“*Make it social”* (48): People can be influenced by their fellow human beings. This must be considered when nudging. Relying on social norms or networks has a positive effect on the nudge. Joining the group in MS Teams where they can chat and see the names of other group members could have create a sense of belonging. So, the context in which the nudges were placed in this study made it social.

“*Make it timely”* *(**48**):* Putting the nudge at the right time and place is just as important as designing the nudge (49). The nudges were broadcast during the morning. This time was chosen because it is assumed that most students study in the morning or just before noon. In addition, students who start later could still view the nudge and did not miss anything. This way it could be ensured that the nudges reached the students in any case.

#### MICG

To determine the effect of digital nudging in the context of PA breaks there was a MICG with access to ESD *via* one-time provision of the link to the videos ESD before the intervention phase. Participants of the MICG did not get further direct reminders during the intervention period, so there was free self-determined decision-making regarding the implementation of ESD.

### Measures and covariates

#### Primary outcome: Daily participation in PA breaks

Each day, daily participation behavior was assessed retrospectively in the evening during the intervention phase (*t*_1_-*t*_10_) by asking participants about the amount of time (in min) they had spent on PA. Therefore, there was an option to answer, “I participated in the Bewegungssnack digital” within the overarching question “How much time did you spend on physical activity today and in what context.” This form of questioning was based on Feuerhahn et al. (50), who used it to capture time spent on leisure activities in their day-level study. It was also adapted for the other day-level questions in the present study.

For the primary outcome of the study, the daily question about ESD participation was included in the analysis as a dichotomous variable. If someone had indicated at least 1 min for participation in the ESD, it counted as participation in the ESD (yes). This was content-related, as there are currently no recommendations on the frequency or duration of breaks in sedentary behavior due to limited evidence (17). From a statistical perspective, this overcame the much-skewed distribution due to the dominant zero-min responses for participation in the ESD. To characterize both groups, the percentage of participants in each group per day (at least 1 min a day) is presented in Table 1.

**Table 1:** Description of the variables included in the analysis for both groups (IG, intervention group; MICG, minimal intervention control group).

	**Variables**	**IG (*n* = 22)**	**MICG (*n* = 35)**
Primary outcome	**Physical activity break**		
	**Exercise snack digital:** Average percentage of participants in the group per day (at least 1 min a day)	60.71%	49.19%
Level 1	**Day-level learning behavior variable**		
	**Home Studying:** *M* (SD) Average sum of hours per day	4.78 (1.46), Range 2.79–8.33	4.45 (1.82), Range 1–9.70
	**Day-level PA behavior variable**		
	**PA total:** *M* (SD) Average sum of min per day	53.64 (35.12), Range 6.67–157.00	55.00 (33.63), Range 0–177.78
	**Day-level variable on the day of the week**		
	**Weekday:** Workday vs. weekend day	8 vs. 2	8 vs. 2
Level 2	**Main characteristic (intervention)**		
	**Group**	*n* = 22	*n* = 35
	**Other co-founders (surveyed at the beginning of the study in T1)**		
	**Age** *M* (SD)	23.95 (2.67), Range 19–32, (*n* = 21)	23.24 (2.89), Range 18–29, (*n* = 33)
	**Previous BSD participation**	Yes: 5, 23.8%	Yes: 4, 12.1%
	**Recommendations fulfilled**	Yes = 7, 33.3% (*n* = 21)	Yes = 17, 51.5% (*n* = 33)

#### Independent variables

In addition, secondary effects that might influence participation behavior in PA breaks were considered. Therefore, PA behavior was taken into account in multiple ways: namely, the PA level of the participants before the intervention in relation to the recommendations for health enhancing PA, and also the time spent on PA outside of PA breaks on a day during the intervention period. Additionally regarding study behavior, the daily time spent on home studying was considered, as longer time spent on working or home studying results in a higher need for breaks regarding the psychological detachment in leisure time (50). Also, the kind of day in the intervention period (work or weekend days) was added as a confounder. In the case of sociodemographic factors, due to the empirical situation, only age is added to the study.

The previous participation in ESD was assessed before the intervention phase (T1) by asking the question: “Do you currently participate in the Bewegungssnack digital?” The covariate was included in the analysis as a dichotomous variable by summarizing both answer categories “irregularly” or “regularly” to represent ESD participation before intervention with respect to the answer “no,” which represents no participation before.

Since the willingness and/OR even the need for a PA break might depend on the one's PA level, the PA level of the subjects was recorded before the intervention in relation to the recommendations for health enhancing PA. Therefore, the Physical Activity Questionnaire of the European Health Interview Survey (EHIS-PAQ) was used at T1 (51). The questionnaire allows for the differentiation of individuals based on whether or not they meet current recommendations for health-enhancing PA (51). Hence, this variable was included as a binary variable in the analysis.

Furthermore, the time spent on PA besides ESD was recorded on daily level. Therefore, the collection of time spent on other PA on a daily level was integrated into the overarching question “How much time did you spend on physical activity today and in what context,” which was also used to measure the daily participation in ESD (see above). Participants could report their time in min for four different forms of PA: two variables for structured supervised exercise (1. university sports courses and 2. other organized sports activities) and two variables for completely self-organized PA (3. independent PA at home like a workout or similar vigorous activity such as cleaning or tidying up and 4. independent PA outside like walking, cycling, jogging, a workout or similar). A total PA score was constructed across the different activity types according to the different domains of health enhancing PA (18). Therefore, the reported min of all four forms of PA were summed up. The total PA score was included in the analysis as a metric variable in hours per day.

Further, the amount of time (in hours) spent studying at home each day was assessed. Following the question type of Feuerhahn et al. (50) participants were asked “How much time did you spend on home studying today.” The time spent on home studying was included in the analysis as a metric variable in hours per day. In addition, the distinction between weekday and weekend was considered as a confounding variable. Since the intervention lasted a total of 10 days and included a weekend on the fourth and fifth day, the two categories have a ratio of 8–2. Age is included as a sociodemographic factor metric variable. Age was asked in number of years in whole numbers before the intervention at T1.

#### Manipulation check

Participants of both groups were asked about how they judge the daily messages in the survey after the intervention phase (T2). Since only the IG received explicit daily nudges and the MICG only had low-threshold indirect reminders regarding the daily surveys, each group had a different reference to the daily messages which they judge. The response had to be given on response options between opposite adjective pairs (scaled 1–4), with higher values representing more positive judgements. The following pairs of opposites were asked: “disturbing” vs. “not disturbing”, “not motivating” vs. “motivating”, “not informative” vs. “informative”, “not appealing” vs. “appealing”, and “restricting” vs. “not restricting”. The ratings of the daily messages revealed no significant group differences for the categories “not disturbing”, “informative”, and “not restricting” (Table 2). However, the IG significantly rated the daily messages much more positively than the autonomous group regarding the categories “motivating” and “appealing” (motivating: *M* = 2.40, SD = 1.10 vs. *M* = 1.76, SD = 0.87, *p* < 0.05; appealing: *M* = 3.05, SD = 0.83 vs. *M* = 1.85, SD = 0.71, *p* < 0.01).

**Table 2:** Results of the manipulation check regarding the rating of the daily messages during the intervention phase for both groups (IG, intervention group; MICG, minimal intervention control group).

	**Variables**	**IG (*n* = 20)**	**MICG (*n* = 33)**	**Group comparison**
	**(range 1–4)**	***M* (SD)**	***M* (SD)**	**(Mann-Whitney-*U*-Test)**
Manipulation check	**Answer of the question “How do you judge the daily messages?” (surveyed at the end of the study in T2)**			
	Disturbing–not disturbing	3.10 (1.12)	2.70 (1.05)	*U* = 256.00, *p* = 0.16
	Not motivating–motivating	2.40 (1.10)	1.76 (0.87)	*U* = 218.00, *p* = 0.03
	Not informative–informative	2.80 (0.95)	2.42 (0.97)	*U* = 260.50, *p* = 0.18
	Not appealing–appealing	3.05 (0.83)	1.85 (0.71)	*U* = 99.50, *p* < 0.01
	Restricting–not restricting	2.85 (1.04)	2.48 (1.09)	*U* = 268.50, *p* = 0.24

### Statistical analysis

Descriptive and inferential statistics were performed by utilizing SPSS 25 (IBM) and R (version 4.1.2). For reasons of comparability, all metric independent variables were z-standardized before the analyses.

#### Main analysis

For the main analysis, logistic regressions were computed to predict participation in ESD. Empirical testing of the theoretical considerations requires models in which individual characteristics are nested within group-related characteristics. For the justification of a multilevel approach, the intercept-only model for the participation behavior across both groups was set up to determine the influence of the intervention on the participation behavior in the ESD as a whole. The intercept-only model suggests the use of multilevel modeling because the intraclass correlation of 0.86 was much higher than 0.10. Therefore, for the main analysis, two-level binary logistic regression models were calculated using the package lme4 with the glmer-function in the following steps (52).

To answer the main research question, at the first step the group variable was included in the intercept model (*Baseline Model*), which contains only the main effect. It determines the influence of the group assignment on the ESD participation behavior.

Second, the Baseline Model was compared to the *Full Model*, which includes all variables with possible secondary effects. In terms of secondary effects, all parallel mechanisms considered that might influence ESD participation behavior were added to the Baseline Model at once. So, besides “group,” the Full Model also contained the group-related characteristics that can differ between individuals; “fulfillment of PA recommendations”, “age” (in years), and “previous participation in ESD” (level 2) as well as individual characteristics that can change within individuals; “daily time for home studying” and “daily time for PA” and the variable of “work and weekend days” (level 1).

Third, from all possible combinations of adding variables regarding secondary effects to the Baseline Model, the model that best predicted daily ESD participation (*Best Model*) was determined. To do this, we compared models with all remaining possible combinations of variable composition that contain the main feature “group” (besides the Baseline Model and the Full Model, 61 models remained). As criterion for model selection, Bayesian information criterion (BIC) was used. It is applied for model selection to address which model generated the data. Smaller values indicate better models (53).

For all models, fixed effect estimates and their corresponding *p*-values were considered for statistical significance. In addition, odds ratios (OR) were calculated as effect sizes. The dataset had up to 12% missing values. To consider the influence of these missing values, a sensitivity analysis was performed in which the main analyses were recalculated using imputed datasets. Therefore the missing data were estimated using the method of multiple imputation within the statistical program R and the mice (multiple imputation by chained equations) packages (54). The process of multiple imputation was computed by creating 27 datasets according to the package howManyImputation based on Von Hippel (55). Across all the datasets, non-missing values are the same, but with different plausible values for missing values. The main analyses ran based on each of the 27 datasets and pooled the estimates together with the additional broom package (56) to get average regression coefficients and correct standard errors. Results were compared regarding fixed effects estimates, their corresponding *p*-value and odds ratios. As BIC-values could not be calculated based on imputed datasets with the current technical standard, this analysis was only based on the datasets with missing values. The comparison between the main analysis and the sensitivity analysis run with imputed datasets showed no large differences and resulted in the same statistically significant findings. Results and a more detailed description of the imputation process can be found in the Appendix (Supplementary Table 1).

## Results

Information on characteristics of both study group were presented in Table 1. There were no significant differences between the two study groups regarding any of the included variables. For the primary outcome the average percentage of participants who performed ESD per day were compared between both groups. While in the IG the percentage of participants per day was 60.7%, in MICG it was 49.2%.

For the main analysis, the results of three binary logistic models are presented in Table 3.

**Table 3:** Overview of the results of the binary logistic structural hierarchical model for the Baseline Model, Full Model, and Best Model.

		**Term**		**Baseline Model**	**Full Model**	**Best Model**
		(Intercept)	β (std. error)	−0.04 (+/−0.45)	0.15 (+/−0.75)	0.04 (+/−0.54)
			*t*	−0.10	0.20	0.08
			*p*	0.92	0.84	0.94
			OR, 95% CI	0.06 [0.40, 2.31]	1.17 [0.27, 5.07]	1.04 [0.37, 2.97]
Level 2	1	Group	β (std. error)	0.74 (+/−0.72)	0.75 (+/−0.90)	0.69 (+/−0.86)
			*t*	1.02	0.83	0.81
			*p*	0.31	0.41	0.42
			OR, 95% CI	2.09 [0.51, 8.55]	2.11 [0.36, 12.4]	2.00 [0.37, 10.8]
	2	Fulfillment of PA recommendations	β (std. error)		0.25 (+/−0.90)	
			*t*		0.28	
			*p*		0.78	
			OR, 95% CI		1.28 [0.22, 7.51]	
	3	Previous ESD participation	β (std. error)		−0.10 (+/−1.21)	
			*t*		−0.08	
			*p*		0.94	
			OR, 95% CI		0.91 [0.09, 9.72]	
	4	Age^*a*^	β (std. error)		−0.25 (+/−0.43)	−0.24 (+/−0.42)
			*t*		−0.58	−0.58
			*p*		0.56	0.57
			OR, 95% CI		0.78 [0.33, 1.80]	0.79 [0.34, 1.79]
Level 1	5	Daily home study hours^a^***	β (std. error)		1.07 (+/−0.18)	1.11 (+/−0.18)
			*t*		5.91	6.27
			*p*		p < 0.001	p < 0.001
			OR, 95% CI		2.93 [2.05, 4.18]	3.03 [2.14, 4.28]
	6	Daily PA total hours^*a*^	β (std. error)		−0.04 (+/−0.16)	
			*t*		−0.26	
			*p*		0.79	
			OR, 95% CI		0.96 [0.71, 1.30]	
	7	Workday	β (std. error)		−0.29 (+/−0.34)	
			*t*		−0.86	
			*p*		0.40	
			OR, 95% CI		0.75 [0.38, 1.46]	
			BIC	540.9	484.3	461.9
			Random effects: SD of	2.42	2.82	2.81
			part–id intercept

^***^High significant; OR, odds ratio; conf. low-conf. high, endpoints of the 95% confidence interval; SD, standard deviation.

PA, physical activity; ESD, exercise snack digital (name of the PA break videos).

^*a*^These variables were z-transformed.

The Baseline Model (BIC = 540.9) showed no significant influence of the group variable on participating in ESD (β = 0.74, *p* =0.31, OR = 2.09).

The Full Model (BIC = 484.3) revealed that only the variable daily home study hours had a significant influence on performing an ESD (β = 1.07, *p* < 0.001). The odds ratios from this variable (OR = 2.93; 95% CI [2.05, 4.18]) indicated that an increase of one standard deviation (SD = 1) in daily home study hours improved the probability of participating in ESD by a factor of 2.93. One standard deviation equals 1.67 hours of daily home study. The estimate for the group variable showed a similar value as in the Baseline Model, but still missed the significance level (β = 0.75, *p* = 0.41, OR = 2.11; 95% CI [0.36, 12.4]).

The Best Model (lowest BIC = 461.9) also contained the variable “age” and the variable “daily home study hours”. Again, only the variable of daily home study hours was significant (β = 1.17, *p* < 0.001). The odds ratio from daily home study hours (OR = 3.03; 95% CI [2.14, 4.28]) indicated that a one standard deviation (SD = 1) increased in the variable improved the probability of participating in ESD by a factor of 3.03. The estimate of the variable group slightly lowered and still showed no significant effect (β = 0.69, *p* = 0.42, OR = 2.00; 95% CI [0.37, 10.8]).

## Discussion

This present study gives insights on how to reach university students in order to promote PA breaks during home study time. Considering the fact that academic studies often impose high demands on university students, PA breaks during a long day of home studying are of particular importance. In conjunction with the COVID-19 pandemic, higher screen time led to an increase in sedentary behaviors among students (6, 7), while PA among young adults was observed to decrease compared to pre-pandemic levels (19–23). Therefore, PA breaks have been identified as promoting factors for university students' physical and mental health. Thus, it is important to investigate how PA breaks can be promoted for students—especially when they are studying at home.

The purpose of this study was to test the effectiveness of digital nudging using a randomized intervention design including an IG who received daily digital motivational prompts for PA breaks. This kind of digital nudging intervention was compared to a minimal control intervention in which participants only got low-level access to PA break *via* a one-time sent link to a media library. The main hypothesis of the present study was not confirmed by binary logistic hierarchical models. The digital nudging condition including daily motivational prompts did not significantly increase the probability of participating in ESD. Neither of the calculated models revealed a statistically significant group effect. Being part of the IG improved the probability of participating in ESD by a factor around of two. It was therefore the second highest estimate in the model, whereby the confidence intervals of the parameter estimates indicate a high uncertainty regarding the true effect. Thus, the digital nudging intervention did not show any significant effect on the likelihood of participating in ESD during a given day during the intervention period. This remains the case, even as the daily nudging messages were rated motivating and appealing by the participants. Previous research has shown that appealing to intentions is an effective way of nudging (34). Providing health benefits or disclosing risks also motivates people to reflect on and change existing behaviors (33, 34). However, the positive results in the manipulation check in the present study were not accompanied with statistically significant effects on PA break behavior of the university students. Here, it is important to consider that the MICG also received some low-threshold, indirect reminders through the daily surveys, although these messages were not as positively rated as the direct reminders, including the digital nudges. Either way, this could have evoked self-reflection which might have triggered behavioral change. This would be in line with results of studies using smartphone apps, where daily self-reports of health behaviors collected *via* the smartphone supported reductions in sedentary behavior (57). Similarly, regarding fitness trackers, Kocielnik et al. (58) has shown that mini-dialogues based on reflecting questions were successful in triggering reflection, resulting in increased motivation, empowerment, and the adoption of new behaviors. Another reason as to why the main hypothesis was not confirmed may be the small number of study participants. Additionally, the daily surveys were not answered by every student each day, resulting in missing data. Thus, the rejection of our main hypothesis could be due to reasons of statistical power which limited the precision of the effect estimates indicated by the rather large confidence intervals of the odds ratio for group effects. Further studies with larger sample size are necessary in order to evaluate whether the observed point odds ratio estimate favoring the digital nudging intervention in this study could be replicated for similar types of interventions and with more precise effect estimates. Furthermore, it is not known what impact the COVID-19 pandemic had on the outcome of the study. Because the COVID-19 pandemic negatively affected the mental health of university students and led to an increase in mental disorders among students (59, 60), mental health problems may have negatively affected participation behavior, regardless of the nudging condition. Therefore, further studies with similar intervention during periods without pandemic constraints are needed. In contrast to the present study, Robroek et al. (37) found a positive effect of monthly email nudging on the likelihood of visiting a health-promoting website offered but not of using the web-based tools. This was however based on two follow-up questionnaires each after 1 year, rather than daily questionnaires as in the present study. It might be that the duration of the intervention also effects the effectiveness of the digital nudging intervention. According to Wood and Neal (61), behavior change interventions of longer duration tend to be more successful in enabling the formation of new habits. However, with longer durations there is a risk that people will get used to nudging and will therefore no longer notice it (62). Landais et al. (32) noted in their review of choice architecture interventions to change PA and sedentary behavior that only one study which prompted PA through email by emphasizing health benefits reported effectiveness of digital nudging (41), and another one mixed effectiveness (42). Regarding reduction of sedentariness, they also pointed out that one study showed effectiveness by prompting PA breaks through mobile phone messages (35). The results of the present study added further insights on effectiveness of daily nudging during home studying to this very small and still unclear set of studies. Moreover, the present study also extended the scope to the university setting and to person-specific side effects.

Instead of a group effect, an individual-level effect was the result: the probability of whether an ESD was carried out in a given day improved with an increase in daily time spent on home studying. This effect was the only one in terms of secondary effects considered as parallel mechanisms during the intervention period, and was found consistently in the different models calculated in this study. An increase of 1.67 h (1 SD = 1.67 hours) in daily Home Study hours, improves the probability of participating in ESD by a factor around of three. One explanation for this could simply be based on probabilities: The longer a student studies at home in a day, the more likely he or she is to make a break. This, in turn, may also provide a theory-based explanation for this effect. According to the effort-recovery approach, stressors that occur as a result of studying at home drain resources that can be replenished through recovery measures (63). Consequently, longer home study time results in higher resource demands. In line with psychological detachment from work (50), PA breaks can foster recovery. They might have the potential to restore affective and energetic resources, and to reduce negative mood built up while studying at day level (64). The significant effect of the amount of home study hours on the probability to do an PA break could be thus explained, as students need to restore their affective and energetic resources. However, it should be noted that participating in ESD was counted as at least 1 min per day and no statement can be made here about the health effects compared to non-participation. With regard to the PA recommendations of the WHO (17), in which on the one hand every movement counts, and on the other hand, sitting times are to be broken up, every PA break could be seen at least as a first step in line with current PA recommendations.

### Strengths and limitations

Certain limitations must be considered when interpreting the results. Regarding the gender distribution of the convenience sample of the study, there is a shift toward more female students (11.1–87.0%). Thus, possible sampling bias cannot be ruled out. Accordingly, generalizability of the associations would still need to be empirically verified.

Furthermore, the overall small sample size comprises a short-coming of the present study. In addition, the sample size of both groups was not equal due to an additional organizational step which only participants of the IG had to carry out. Thus, there may have been a selection effect for the IG, as the less willing group members may have dropped out before the intervention began.

Additionally, the dataset had up to 12% missing values, which produced a lack of information. If there is no answered questionnaire from a student, nothing is known about the reasons why. The missing information or impossible assignment could result from the fact that the student refused to participate in the survey, missed or forgot it, that there were technical problems, or that their code deviated from the code of the other survey time points and could not be determined exactly even by means of the manual assignment procedure. Multiple imputation can estimate missing values, but variances remain. However, as results of the three models with imputed datasets show the same statistically significant findings as the results with the dataset with missing values, biased results due to the absence of answered daily questionnaires can be neglected.

Due to the study design with daily surveys during the intervention phase, there is no clear control group without any intervention. There is only a control group with minimal intervention *via* daily surveys. As daily surveys can evoke self-reflection, which might trigger behavioral changes (57), the difference between both groups regarding reminders for participation in ESD might be too small to identify an effect. Accordingly, the results refer only to the comparison between a direct and an indirect form of reminder. In addition, the direct digital nudging of the intervention group may not have been intrusive enough to serve as a stimulus. It may not have attracted enough attention to encourage students to take a PA break.

However, the study design also provides advantages regarding the individual characteristics which can change within a person over the intervention phase, and which are nested within group-related characteristics that differ between individuals. Combined with a multilevel approach by conducting two-level binary logistic hierarchical models, the present study also extended results on person-specific side-effects. Thereby, not only the group difference but also the individual level was considered. The *ICC* = 0.86 of the Intercept Model showed that the person-specific proportion of the probability of whether or not an ESD was carried out on a day is relevant to consider in such an analysis. Up to now, however, most studies have only observed the difference based on pre and post survey dates or data between different treated groups (39, 40, 42). Regarding other nudging studies promoting PA in sedentary working settings, to the authors knowledge only Robroek et al. (37) had conduct using multilevel general estimating equations to identify characteristics of employees who participated in the health enhancing program. But this was based on two follow-up questionnaires each after one year and not referencing daily questionnaires like the present study did.

## Conclusion

Not much is known yet about how PA breaks have been implemented under circumstances of COVID-19 pandemic or how they can be delivered to students when they are not on-site at the university. Therefore, the present study tested digital nudging as an approach to encourage students to take a PA break. However, digital nudging, as conducted in the present study, did not result in a higher likelihood of taking a PA break among the group that received the nudging. The present study did extend results on person-specific side-effects during the nudging on a daily basis. This is important especially in the context of home studying under the containment measures associated with the COVID-19 pandemic, which led to increased mental health problems in addition to increased physical inactivity and prolonged sitting. Since daily home studying time is critical to the likelihood of participating in ESD, it should be taken into account when comparing different nudging interventions in the university setting. The longer students spend daily time for home study, the more likely they are to participate in ESD. Restoration of affective and energetic resources could be a reason for the higher likelihood of participation. The content design of the nudges could benefit from this knowledge by including this possible reason for participating in a PA break in the prompt messages.

Daily digital nudging for digital PA breaks were found to be more motivating and engaging than daily mails in the evening for the survey regarding participation behavior. However, this did not result in statistically significant higher participation behavior in ESD. Larger samples and clear differences in intervention should be attempted to be implemented in further studies. However, potential strategies for interrupting sedentary behavior and introducing PA breaks should not rely solely on the digital nudging. PA breaks integrated into home study lessons *via* presented videos by the lecturer may be another possibility in terms of digital semesters.

## Data availability statement

The raw data supporting the conclusions of this article will be made available by the authors, without undue reservation.

## Ethics statement

The studies involving human participants were reviewed and approved by Ethics Committee of the Faculty of Social Sciences and Economics, University of Tübingen. The patients/participants provided their written informed consent to participate in this study.

## Author contributions

MT, JM, and GS designed the study. MT and JM coordinated and carried out participant recruitment and data collection. MT and DL conducted the data analyses. MT drafted the initial version of the manuscript. All authors contributed to reviewing and editing the manuscript and have read and agreed to the published version of the manuscript.

## Funding

This research was funded by the Techniker Krankenkasse, health insurance fund.

## Conflict of interest

The authors declare that the research was conducted in the absence of any commercial or financial relationships that could be construed as a potential conflict of interest.

## Publisher's note

All claims expressed in this article are solely those of the authors and do not necessarily represent those of their affiliated organizations, or those of the publisher, the editors and the reviewers. Any product that may be evaluated in this article, or claim that may be made by its manufacturer, is not guaranteed or endorsed by the publisher.
