# Supplementary material for: Nudging digital physical activity breaks for home studying of university students—A randomized controlled trial during the COVID-19 pandemic with daily activity measures
Source: Front Sports Act Living. 2022 Nov 24;4:1024996. doi: 10.3389/fspor.2022.1024996 (PMC9729792; doi:10.3389/fspor.2022.1024996)
Supplement: Supplementary file 1 [file Data_Sheet_1.PDF]

# *Supplementary Material*

## 1 Supplementary Figures

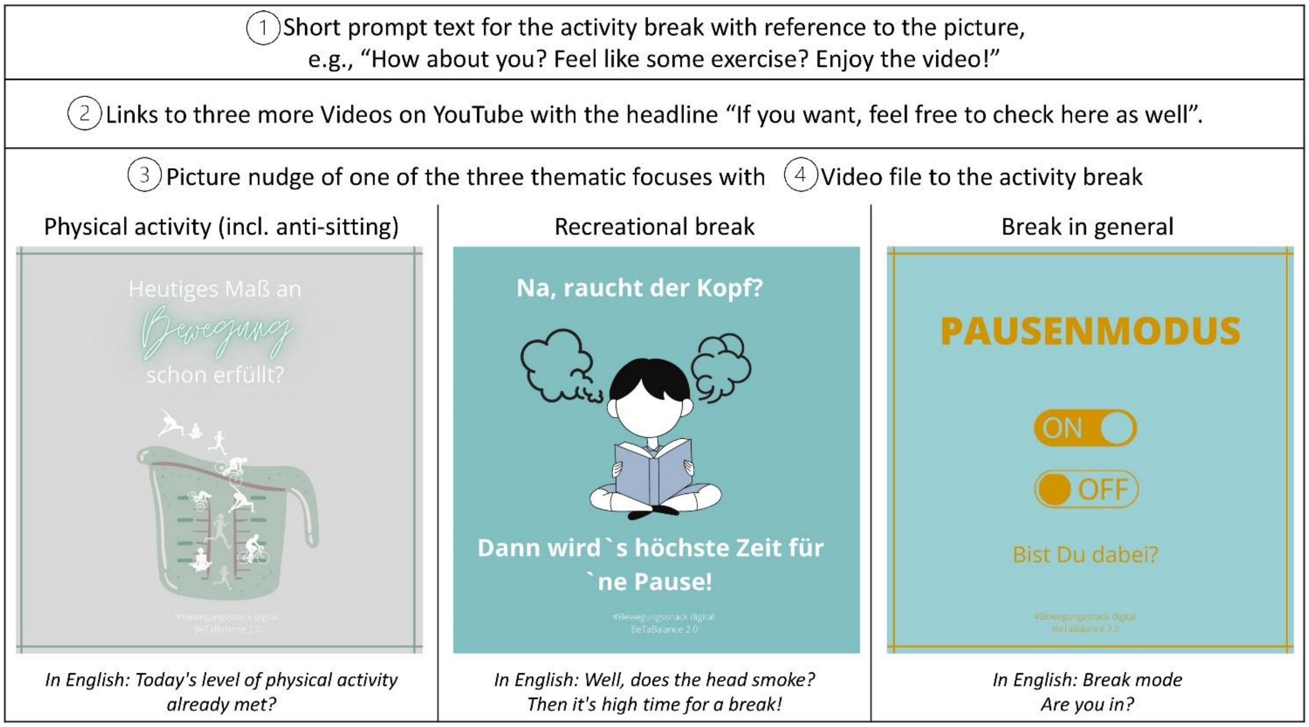


**Supplementary Figure 1.** Structure of the daily nudge which participants of the intervention group (IG) received, based on picture examples from the three categories according to their thematic focuses.

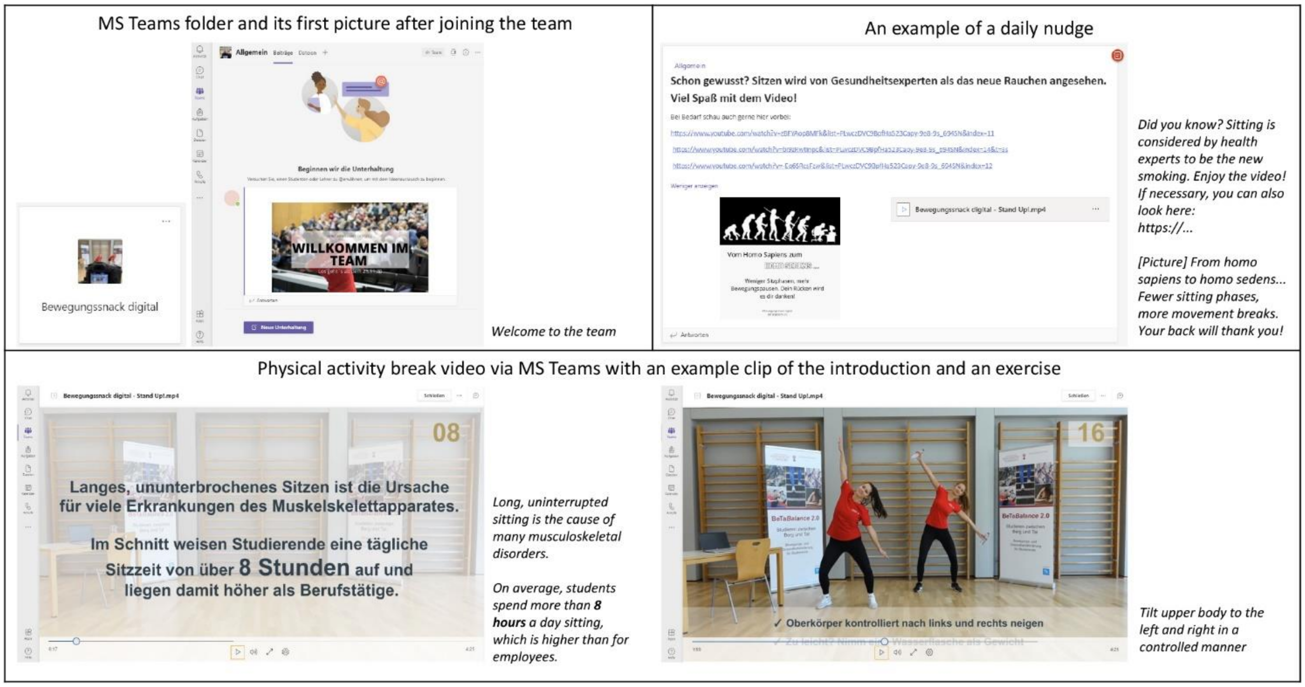


**Supplementary Figure 2.** Example of the team folder, which participants of the intervention group (IG) have joined.

## 2 Supplementary Tables

**Supplementary Table 1.** Overview of the results of the binary logistic structural hierarchical model for the main analysis and the sensitivity analysis for the Baseline Model, Full Model, and Best Model.

| | | | | Baseline Model | | Full Model | | Best Model | |
|---|---|---|---|---|---|---|---|---|---|
| | | Term | | with missings | with 27 imputation[1] | with missings | with 27 imputation[1] | with missings | with 27 imputation[1] |
| | | (Intercept) | β (std. error) | -0.04 (+/-0.45) | -0.01 (+/-0.38) | 0.15 (+/-0.75) | 0.21 (0.05) | 0.04 (+/-0.54) | 0.02 +/-0.39) |
| | | | *t* | -0.10 | 0.04 | 0.20 | 0.39 | 0.08 | 0.04 |
| | | | *p* | 0.92 | 0.99 | 0.84 | 0.700 | 0.94 | 0.96 |
| | | | *OR* | 0.06 | 0.99 | 1.17 | 1.23 | 1.04 | 1.19 |
| Level 2 | 1 | Group | β (std. error) | 0.74 (+/-0.72) | 0.57 (+/-0.60) | 0.75 (+/-0.90) | 0.60 (0.15) | 0.69 (+/-0.86) | 0.53 (+/-0.62) |
| | | | *t* | 1.02 | 0.95 | 0.83 | 0.93 | 0.81 | 0.86 |
| | | | *p* | 0.31 | 0.34 | 0.41 | 0.35 | 0.42 | 0.39 |
| | | | *OR* | 2.09 | 1.78 | 2.11 | 1.82 | 2.00 | 1.71 |
| | 2 | Fulfillment of PA recommendations | β | | | 0.25 (+/-0.90) | 0.18 (0.07) | | |
| | | | *t* | | | 0.28 | 0.28 | | |
| | | | *p* | | | 0.78 | 0.78 | | |
| | | | *OR* | | | 1.28 | 1.20 | | |
| | 3 | Previous ESD participation | β | | | -0.10 (+/-1.21) | -0.25 (0.16) | | |
| | | | *t* | | | -0.08 | -0.32 | | |
| | | | *p* | | | 0.94 | 0.75 | | |
| | | | *OR* | | | 0.91 | 0.78 | | |
| | 4 | age[2] | β | | | -0.25 (+/-0.43) | -0.10 (0.14) | -0.24 (+/-0.42) | -0.08 (+/-0.31) |
| | | | *t* | | | -0.58 | -0.33 | -0.58 | -0.27 |
| | | | *p* | | | 0.56 | 0.74 | 0.57 | 0.79 |
| | | | *OR* | | | 0.78 | 0.90 | 0.79 | 0.92 |
| Level 1 | 5 | Daily home study hours[2] *** | β | | | 1.07 (+/-0.18) | 0.75 (0.33) | 1.1 (+/-0.18) | 0.77 (+/-0.14) |
| | | | *t* | | | 5.91 | 5.50 | 6.27 | 5.67 |
| | | | *p* | | | 3.34e-09 *** | 5.94e-08 *** | 3.66e-10 *** | 2.87e-08 *** |
| | | | *OR* | | | 2.93 | 2.11 | 3.03 | 2.16 |
| | 6 | Daily PA total hours[2] | β | | | -0.04 (+/-0.16) | -0.02 (0.20) | | |
| | | | *t* | | | -0.26 | -0.17 | | |
| | | | *p* | | | 0.79 | 0.87 | | |
| | | | *OR* | | | 0.96 | 0.98 | | |
| | 7 | Workday | β | | | -0.29 (+/-0.34) | -0.31 (0.02) | | |
| | | | *t* | | | -0.86 | -0.97 | | |
| | | | *p* | | | 0.40 | 0.34 | | |
| | | | *OR* | | | 0.75 | 0.73 | | |

[1] The number of 27 imputation was calculated in R with the package how_many_imputations (modelfit1, cv = .1, alpha = .01). Cv and alpha arguments are optional and can be used to tweaked to control how conservative or anti-conversative the estimate is.

[2] These variables were z-transformed.
*OR* = odds ratio; PA = physical activity; ESD = exercise snack digital (name of the PA break videos)

**Method of data imputation:** As the dataset showed missing, the main analysis was run with the original datasets as well as with the imputed datasets to proof the effect of missing data (Supplementary Table 1). Therefore the missing data of the long format dataset with the z-transformed variables were estimated using the method of multiple imputation within the statistical program R and the mice (multiple imputation by chained equations) packages (51). The pattern of missing data of the dataset was explored using the VIM-package (63) to ensure that no variable had more than 25% missings (64). The dataset shows missing data from 0.5 to 0.12 %. In order to proof, how many imputations were needed, the package howManyImputation based on Hippel (2018) were used to calculate the number of imputations that are needed for a final analysis with desired level of replicability (52). It suggested 27 impuation. Metric data were imputed via predictive mean matching (pmm) whereas logistic regression imputation (logreg) was used for binary data. After that, the process of multiple imputation was computed by creating 27 datasets, across all of them, non-missing values are the same, but with different plausible values for missing values. The main analyses ran based on each of the 27 datasets and pooled the estimates together with the additional broom package (53) to get average regression coefficients and correct standard errors. Therefore, the with- and the pool-function of the mice package were used.
